# Personalized goal setting and predictors of functional gains following constraint-induced movement therapy in preschool-aged children with unilateral cerebral palsy

**DOI:** 10.1371/journal.pone.0329002

**Published:** 2025-08-06

**Authors:** Youngsub Hwang, Jeong-Yi Kwon

**Affiliations:** 1 Department of Health Sciences and Technology, Samsung Advanced Institute for Health Sciences and Technology, Sungkyunkwan University, Seoul, Republic of Korea; 2 Department of Physical and Rehabilitation Medicine, Sungkyunkwan University School of Medicine, Samsung Medical Center, Seoul, Republic of Korea; Drexel University School of Biomedical Engineering Science and Health Systems, UNITED STATES OF AMERICA

## Abstract

**Objective:**

This study aimed to identify caregiver-selected goal characteristics that predict functional improvements following constraint-induced movement therapy (CIMT), offering novel insights into personalized rehabilitation for younger children with unilateral cerebral palsy (UCP).

**Methods:**

This study included 19 children with UCP aged 4–6 years who participated in a three-week CIMT program comprising 15 sessions (30 hours total), during which the unaffected hand was constrained to encourage intensive use of the affected limb. Caregivers identified five meaningful rehabilitation goals per child using the Canadian Occupational Performance Measure, categorizing them into self-care, productivity, or leisure domains and ranking them by importance. Upper-limb function was objectively evaluated using the Assisting Hand Assessment before and immediately after CIMT. Linear regression analyses identified the factors influencing goal selection, and least absolute shrinkage and selection operator regression determined whether prioritized goal types predicted improvements in upper limb function.

**Results:**

Self-care goals were most frequently selected (72.6%), followed by leisure (26.3%) and rarely productivity (1.1%). Leisure goal selection was significantly associated with greater baseline upper limb range of motion and lower baseline occupational performance scores. The higher prioritization of goals involving quiet leisure activities (e.g., arts, crafts, computer play) and dressing tasks (e.g., buttoning, zipping) significantly predicted greater functional improvements post-intervention.

**Conclusion:**

This study provides important new evidence indicating that caregiver-selected rehabilitation goals that are closely aligned with a child’s latent motor capacities positively affect functional outcomes. These findings underscore the clinical importance of individualized, family-driven goal setting for optimizing therapeutic effectiveness in preschool-aged children with UCP.

## Introduction

Cerebral palsy (CP) is a group of permanent disorders affecting the development of movement and posture, attributed to non-progressive disturbances in the developing fetal or infant brain [1]. The clinical presentation of CP is heterogeneous, encompassing several subtypes, including spastic, dyskinetic, ataxic, and mixed types. Among them, spastic CP is the most prevalent, accounting for approximately 80% of cases [2].

Unilateral cerebral palsy (UCP), also known as spastic hemiplegia, is the most common form of spastic CP and is characterized by motor impairments predominantly on one side of the body [3]. The severity of upper-limb dysfunction in UCP varies widely, depending on the timing, location, and extent of the brain lesion [4]. Children with UCP often experience reduced spontaneous use of the affected upper extremity in daily tasks, which may be further compounded by secondary musculoskeletal deformities, abnormal postural control, and impaired sensory and cognitive integration [5–7]. These impairments contribute to significant limitations in participation, prompting extensive efforts by occupational therapists and rehabilitation researchers to develop and evaluate interventions that enhance upper-limb function in this population [8].

Goal setting, which involves identifying functional activities that the child and family prioritize for improvement during therapy, is widely acknowledged as a core component of pediatric rehabilitation, aligning therapy with the child’s and family’s priorities to foster motivation and enhance outcomes [9,10]. Among the available tools supporting this process, the Canadian Occupational Performance Measure (COPM) has gained recognition for its ability to facilitate personalized goal identification and evaluate perceived changes in performance and satisfaction [11]. Grounded in the Canadian Model of Occupational Performance and Engagement (CMOP-E), COPM identifies occupational difficulties across self-care, productivity, and leisure and is widely used as an assessment tool in family centered interventions, providing a structured approach to understanding individualized functional needs [12]. Although this principle is well-established, few studies have explicitly examined how specific goal characteristics relate to meaningful gains in UCP, and even fewer have focused on younger children.Metzler et al. (2021) [13], for instance, investigated school-aged children with UCP and found that those with higher motor abilities tended to select leisure goals, whereas those with lower abilities prioritized self-care. While such studies provide valuable insights, no previous research has specifically investigated how caregivers prioritize rehabilitation goals for preschool-aged children with UCP or examined how these priorities influence functional outcomes after intensive motor interventions.

Preschool-aged children with UCP undergo rapid motor and self-care skill development, playing as fundamental role in fostering their independence and participation [14]. Given their young age, these children often struggle to independently identify and prioritize meaningful therapeutic goals, making the role of caregivers essential in the goal-setting process [15]. Parents are instrumental in establishing rehabilitation priorities and ensuring that the selected goals align with the child’s environment and daily life demands [16]. Furthermore, greater caregiver involvement and understanding have been linked to improved goal attainment, as parents facilitate opportunities for skill development in natural contexts and reinforce therapeutic activities beyond clinical sessions [17]. Consequently, understanding how caregivers select goals and how these choices influence functional progress is essential for refining rehabilitation strategies. Despite the recognized importance of individualized goal setting, there is limited research on goal selection patterns in this younger population, particularly regarding how developmental needs interact with goal priorities and therapeutic outcomes. Investigating these factors can provide crucial insights for optimizing early intervention approaches and ensuring that therapy aligns with the child’s emerging abilities and family priorities.

This study aimed to address abovementioned gaps by characterizing individualized goal-setting preferences in preschool-aged children with UCP, examining the factors influencing their goal choices, and identifying the predictors of functional improvements following intensive intervention. Insights gleaned from the findings could support the development of targeted age-appropriate strategies that optimize participation and foster meaningful progress during the critical stages of child development.

## Materials and methods

### Study design and participants

**Fig 1:**
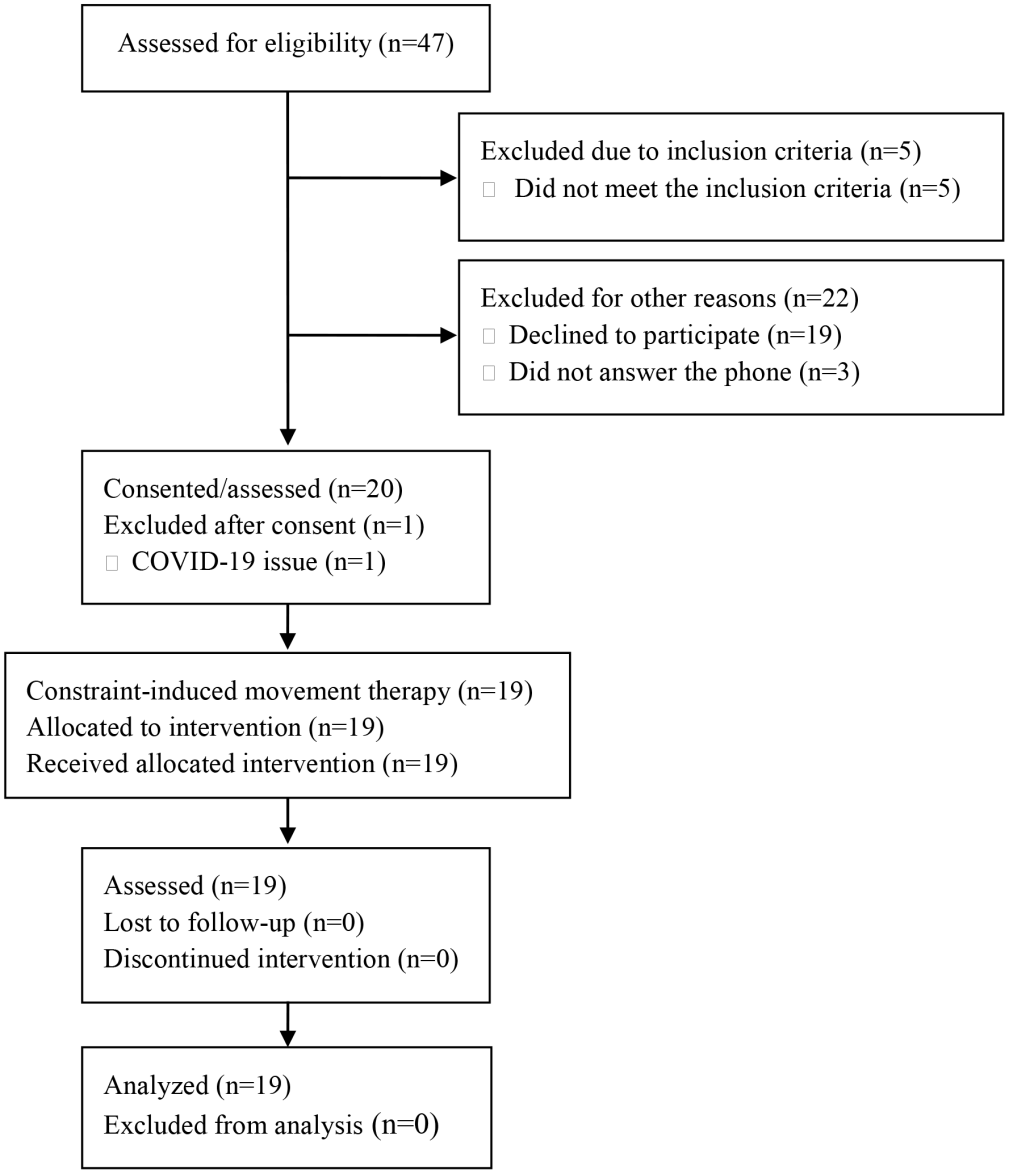
CONSORT 2010 flow diagram. Forty-seven children with unilateral cerebral palsy were initially assessed for eligibility. Of these, 27 were excluded: five did not meet the inclusion criteria, 19 declined to participate for personal reasons, and three were unreachable. Twenty children provided consent and completed the baseline assessments. One participant was subsequently excluded due to COVID-19-related issues prior to intervention initiation. Ultimately, 19 children completed the full constraint-induced movement therapy (CIMT) program and all post-intervention assessments; their data were included in the final analysis.

Children with UCP aged 4–6 years were included in this nested observational analysis, derived from a larger two-phase randomized controlled trial (RCT) conducted from July 2021 to December 2022 (ClinicalTrials.gov identifier: NCT04904796). The original RCT recruited participants aged 4–12 years with lesions in the central nervous system. For this study, however, we specifically analyzed data from the group aged 4–6 years to investigate developmental considerations unique to preschool-aged children. Data were collected at a tertiary hospital in Seoul, Republic of Korea. The exclusion criteria included severe cognitive dysfunction that hindered the ability to perform simple tasks, untreated seizures, visual or auditory issues that could impede treatment, and a history of musculoskeletal disorders. The study was approved by the hospital review board (approval no. SMC 2021-04-042). Written informed consent was obtained from all participants’ parents or legal guardians prior to enrollment, in accordance with the Declaration of Helsinki. Fig 1 illustrates the detailed participant selection process.

### Interventions

The following intervention protocol was implemented as part of the parent RCT (NCT04904796). For the present study, we only analyzed data from participants in the intervention group, without group comparison or randomization-related outcomes. Each participant underwent 15 sessions over three weeks (two hours per session, five sessions per week, totaling 30 hours). A standardized protocol incorporating functional, task-oriented activities and shaping techniques [17] was delivered by trained therapists, who provided graded challenges to encourage targeted movements. A custom detachable forearm splint was worn on the less-affected limb throughout each session, except for brief safety or comfort breaks. While the content of each session was adapted to each child’s abilities, a uniform structure emphasizing the increased use of the more affected limb was maintained.

### Outcomes

Assessments were conducted at two time points: baseline (T0) and immediately after CIMT (T1). The main objective outcome measure was the Assisting Hand Assessment (AHA), which evaluates the spontaneous use of the affected hand during bimanual activities [18]. COPM [19] was the subjective outcome measure used to assess participants’ self-perceived performance and satisfaction across individually identified goals. At baseline, parents collaboratively identified five personally meaningful goals per participant, prioritizing them from most to least important. COPM performance and satisfaction scores were recorded using a 10-point Likert scale (1 = “not able to do” or “not satisfied at all”; 10 = “able to do extremely well” or “extremely satisfied”).

To assess motor function, the Melbourne Assessment 2 (MA2) was used as an objective measure of upper-extremity motor capacity [20]. Additionally, the Pediatric Motor Activity Log (PMAL) was used to evaluate the frequency and quality of use of the affected limb in daily activities [21].

### Classification of goals

Goals identified through COPM were independently categorized by two raters into three domains based on CMOP-E: (1) self-care (activities involving personal care, such as dressing, eating, hygiene, bathing, toileting, and transfers), (2) productivity (activities related primarily to play tasks), and (3) leisure (activities undertaken for enjoyment, subdivided into quiet and active leisure). In cases of discrepancies, a third rater provided input until a consensus was reached. Goal categorization aimed to reflect participants’ individual priorities, developmental levels, and functional abilities.

### Data analysis

Statistical analyses were conducted using IBM SPSS Statistics (version 25.0; IBM Corp., Armonk, NY, USA) and R software version 4.1.0. Descriptive statistics summarized participant demographics and the distribution of COPM goals categorized according to CMOP-E.

To examine the factors influencing COPM goal selection, each participant’s goals were quantified as proportions within the three CMOP-E domains: self-care, productivity, and leisure. For example, selecting two of the five self-care goals resulted in a proportion of 0.4. Owing to the relatively large number of potential explanatory variables compared with our sample size, two-step regression was employed. First, univariate linear regressions were conducted separately for each independent variable group: (1) demographic variables (age, Manual Ability Classification System level, and sex), (2) cognitive measures (FSIQ and subindices), and (3) baseline clinical assessments (AHA, MA2, PMAL, and COPM at baseline). Variables demonstrating statistical significance (p < 0.05) in univariate analyses were subsequently included in the multiple linear regression model, with nonsignificant variables (p > 0.05) sequentially removed. Regression assumptions, including linearity, normality, homoscedasticity, and multicollinearity, were verified and satisfied. Statistical significance was set at p < 0.05.

To determine whether COPM goal rankings predicted improvements in AHA scores following CIMT, multinomial logistic regression was initially used to calculate the rank probabilities for each COPM goal based on prioritization (from rank 1, highest priority, to rank 5, lowest priority). Owing to the high number of predictors relative to the sample size, LASSO regression was performed, as it effectively selects significant variables by penalizing less influential predictors toward zero. Importantly, all available candidate variables—regardless of their statistical significance in univariate analyses—were entered into the LASSO model. We selected this approach to minimize the risk of overlooking potential associations or interactions and to ensure that model selection was not biased by prior variable filtering. The optimal penalty parameter lambda, (λ) selection was determined using tenfold cross-validation repeated 100 times, and the averaged regression coefficients from these repetitions provided stable estimates of significant predictors.

## Results

### Participant characteristics

This study included data from 19 preschool-aged participants (10 boys; mean age: 4.80 ± 0.77 years). Each participant identified five goals using COPM, resulting in a total of 95 goals. Table 1 presents detailed characteristics of the children.

**Table 1:** Participant characteristics.

Child	Sex	Corrected age (y)	Birth term	MACS level	FSIQ
1	Male	5.17	Full	1.00	111.00
2	Female	5.00	Pre	2.00	73.00
3	Male	5.42	Pre	3.00	103.00
4	Male	6.50	Pre	1.00	52.00
5	Female	5.00	Full	1.00	109.00
6	Female	4.42	Pre	1.00	51.00
7	Male	4.33	Pre	3.00	44.00
8	Male	4.08	Full	2.00	117.00
9	Female	4.00	Pre	3.00	–
10	Male	4.00	Full	1.00	72.00
11	Male	4.17	Full	2.00	100.00
12	Female	4.50	Full	2.00	86.00
13	Female	5.08	Full	3.00	105.00
14	Female	5.83	Pre	1.00	73.00
15	Male	4.33	Pre	1.00	123.00
16	Male	6.00	Full	2.00	84.00
17	Male	4.00	Full	2.00	103.00
18	Female	5.42	Pre	2.00	71.00
19	Female	4.00	Full	2.00	–

FSIQ, Full-Scale Intelligence Quotient; MACS, Manual Ability Classification Systemic; -, N/A.

### COPM goal categorization

COPM goals categorized using CMOP-E revealed that the majority of the selected goals belonged to the self-care domain (72.6%), followed by leisure (26.3%), and productivity (1.1%). Within self-care, the most frequently addressed goals were dressing (20.6%), hygiene (16.9%), eating (15.2%), bathing (13.0%), toileting (4.7%), and transfers (2.2%). Leisure activities predominantly involved quiet leisure (24.1%), such as turning pages of a book, threading, origami, and computer usage, whereas active leisure tasks (e.g., catching a ball or jumping rope) were minimal (2.2%). Productivity-related goals were rarely identified, with only one instance (1.1%) related to play activities such as console gaming (Table 2).

**Table 2:** Distribution of goals categorized by CMOP-E (participants chose five goals), with examples from the data.

Goals by category	% of goals by participant (n = 19), range	% of total goals (n = 95)	*Participant example*
**CMOP-E**			
** Self-care**	40–100	72.6	
Dressing	20–40	20.6	Buttoning and unbuttoning shirts, zipping up jackets, pulling sweaters overhead, wearing gloves independently, securely fastening shoes with Velcro or laces
Eating	0–20	15.2	Holding bowls or cups steadily while eating or drinking, lifting a plate, cutting food with a knife, correctly using a fork to eat food with various textures
Hygiene	0–40	16.9	Washing face thoroughly with both hands, independently removing and replacing toothpaste caps, applying an appropriate amount of toothpaste to a toothbrush
Bathing	0–40	13.0	Washing and drying both paralyzed and non-paralyzed limbs effectively, independently washing hair using both hands including applying and rinsing shampoo
Toileting	0–20	4.7	Tearing toilet paper accurately, folding toilet paper appropriately, independently sitting down on and standing up from the toilet seat, safely and without assistance
Transfers	0–20	2.2	Holding securely onto handrails for stability, safely ascending and descending stairs using alternating or step-by-step foot placement
**Productivity**	0–20	1.1	
Play	0–20	1.1	Operating console gaming controllers independently, pressing buttons accurately
**Leisure**	0–60	26.3	
Quiet leisure	0–60	24.1	Independently turning the pages of books (storybooks, picture books) with controlled hand movements, threading beads or string through small holes, creating basic origami figures (e.g., paper animals, boats), precisely applying glue for craft activities, effectively using computer mice and keyboards for educational software and digital play
Active leisure	0–20	2.2	Catching balls of various sizes thrown from short distances, effectively jumping rope with coordinated movements using both hands and feet

CMOP-E, Canadian Model of Occupational Performance and Engagement.

To further explore differences by manual ability, we conducted a subgroup analysis of five frequently chosen goal categories (dressing, hygiene, eating, bathing, and quiet leisure) according to the Manual Ability Classification System (MACS) levels I–II versus level III. There was no evidence of a difference in the MACS III group participants compared with other group participants in dressing (8.20 ± 1.64 vs. 9.00 ± 1.16) and quiet leisure (6.80 ± 1.92 vs. 8.26 ± 1.25) (S1 Table).

### Factors influencing COPM goal selection

Regression analysis was conducted to identify the factors influencing the proportion of goals selected within the CMOP-E domains. Owing to limited variability (only one productivity goal), productivity was excluded from further regression analyses. For leisure goal selection, baseline upper-extremity motor function measured by the MA2 Range of Motion (ROM, coefficient = 0.004, p = 0.029) was positively associated, while baseline COPM performance scores (coefficient = −0.063, p = 0.029) were negatively associated with leisure goal selection. The model demonstrated moderate explanatory power (R² = 0.371; Table 3).

**Table 3:** Regression analysis summary for COPM goal selection.

Variable	Coefficient (estimate)	Std. error	t	p	R²
**Leisure**					0.371
Intercept	0.209	0.139	1.51	0.148	
MA2 ROM (T0)	0.004	0.002	2.356	**0.029** ^a^	
COPM performance (T0)	−0.063	0.027	−2.370	**0.029** ^a^	

^a^Significant difference.

T0: baseline.

Std: standard, MA2: Melbourne Assessment 2, ROM: range of motion, COPM: Canadian Occupational Performance Measure.

### COPM problem list rankings as predictors of AHA improvement

Quiet recreation (coefficient = 1.238, p < 0.001) and dressing goals (coefficient = 0.621, p = 0.011) emerged as significant predictors. Selecting higher-ranked quiet recreation or dressing goals significantly predicted greater improvements in upper extremity function, as assessed by the AHA scores (Table 4).

**Table 4:** COPM problem list rankings as predictors of AHA improvements.

Outcome	Predictor	Coefficient (estimate)	Std. error	t	p	95% CI
**AHA (T1-T0)**	Quiet recreation	1.238	0.200	6.192	**<0.001** ^a^	0.786-1.690
	Dressing	0.621	0.193	3.213	**0.011** ^a^	0.184-1.058

^a^Significant difference.

T0: baseline, T1: post-constraint-induced movement therapy.

Std: standard, CI: confidence interval, AHA: Assisting Hand Assessment.

## Discussion

Consistent with previous research on pediatric goal setting [13], our study found that preschool-aged children with UCP most frequently selected self-care goals (72.6%), followed by;eisure (26.3%) and, to a minimal extent, productivity (1.1%). While Metzler et al. [13] reported that self-care was the most common domain among school-aged children (45.2%), older participants demonstrated higher proportions of productivity (15.6%) and leisure (35.5%) goals. Similarly, Chiarello et al. [16] observed that children under six years of age allocated approximately 70% of their goals to self-care, 8% to productivity, and 22% to leisure, closely mirroring our preschool sample’s distribution. This shift suggests that as children grow older, self-care goals become relatively less dominant, whereas more diverse domains—such as productivity-related tasks and leisure pursuits—begin to assume increased importance. A plausible explanation is that older children face broader role expectations, including academic responsibilities [22] and varied recreational interests [23], leading to goal-setting patterns that encompass multiple occupational domains. By contrast, younger preschool-aged children are typically still focused on mastering foundational self-care abilities, which may explain their higher emphasis on basic functional tasks. These findings underscore the importance of recognizing the evolution of goal-setting priorities with age.

In this study, baseline upper-limb range of motion (ROM) assessed using MA2 and baseline COPM performance scores significantly influenced the selection of leisure-related goals. Specifically, children exhibiting greater upper extremity ROM combined with relatively lower perceived occupational performance were more likely to prioritize leisure goals. A plausible interpretation of this finding is that these children had sufficient motor capacity to attempt more varied tasks but still experienced notable functional challenges, leading caregivers to select leisure goals that were simultaneously engaging and achievable. Importantly, these results are consistent with previous findings indicating that improvements in motor capacity do not always directly translate into changes in objectively measured motor performance [24,25]. This underscores the need to design interventions to bridge the gap between capacity and daily performance. In practice, therapists should focus not only on enhancing motor abilities in controlled therapy settings but also on facilitating the integration of these skills into meaningful everyday contexts. By selecting leisure goals that are engaging yet aligned with a child’s functional profile, occupational therapists can help ensure that new motor capacities translate more readily into practical gains in participation, ultimately supporting both immediate skill acquisition and long-term functional independence.

We found that caregiver-prioritized goals related to quiet leisure and dressing were significantly associated with greater improvements in bimanual function, as measured by AHA following CIMT. Given that CIMT primarily targets unilateral motor function, this finding may reflect underlying child characteristics, rather than direct bimanual task engagement, that predispose them to benefit from targeted training. Specifically, caregivers who selected quiet leisure and dressing may have perceived these activities as challenging yet feasible, indicating that their children had sufficient motor capacity but struggled with functional execution.

This interpretation aligns with Chiarello et al. [16], who reported that dressing was chosen by 52% of children in Gross Motor Function Classification System (GMFCS) level I, 36% in levels II/III, and 27% in levels IV/V, implying that those with a lower severity of impairment are more likely to prioritize Dressing goals. Quiet leisure tasks (e.g., threading, origami, and computer use) and Dressing tasks (e.g., buttoning and zipping) require dexterity, fine motor coordination, and controlled hand movements, which could be positively influenced by CIMT’s intensive practice components.

There was no evidence of a difference in the mean importance ratings for dressing (8.20 ± 1.64 vs. 9.00 ± 1.16) and quiet leisure (6.80 ± 1.92 vs. 8.26 ± 1.25) between children classified as MACS level III and those in levels I–II (S1 Table), and the small sample size suggests that any observed differences could be attributable to chance. This aligns with the ongoing uncertainty regarding the influence of impairment severity on CIMT outcomes, as highlighted by Eliasson et al. [26]. Although several studies have included children across various severity levels, the field still lacks a definitive criterion for predicting which subgroups will respond most favorably [27]. Consequently, our results should be interpreted with caution, particularly when extrapolating to children with more pronounced motor impairment. Further research is needed to clarify whether caregiver-driven goal selection can reliably predict responsiveness to CIMT across the severity spectrum in children.

Our findings suggest that when caregivers prioritize developmentally appropriate and moderately challenging goals, such as dressing or quiet leisure activities, the child is more likely to demonstrate functional improvements following CIMT. This observation aligns with established goal-setting theories, which emphasize that goals should balance difficulty and achievability to enhance motivation, engagement, and performance [10]. Therapists can therefore play a critical role in helping families to formulate goals that reflect the child’s latent motor abilities and evolving capacities. From a practical perspective, this insight reinforces the value of structured, family-centered goal-setting frameworks in pediatric rehabilitation. By providing caregiver education and decision-support strategies—particularly in community-based settings—clinicians may extend the benefits of individualized goal setting beyond specialized centers, increasing equity in access to effective early interventions for children with UCP in under-resourced environments. Future implementation studies should investigate how these approaches can be effectively operationalized across diverse care settings.

### Limitations

This study has several limitations. First, the relatively small sample size might restrict the generalizability of the findings and reduce statistical power. However, the focused nature of the target population—preschool-aged children with unilateral spastic CP undergoing CIMT—and our rigorous analytical approach, which included LASSO regression with cross-validation, helped mitigate some of these concerns. Second, goal-setting involved significant input from parents, which, while clinically realistic, might have shifted goal priorities away from the child’s emerging preferences. Third, variations in baseline functional levels and comorbidities were not fully explored in the subgroup analyses, potentially obscuring nuanced patterns of goal selection and outcomes. Finally, all participants were drawn from a single cultural and ethnic background, which may limit the applicability of the results to other populations. Future studies with larger, more diverse cohorts and robust experimental designs are needed to clarify these preliminary findings and refine goal-setting strategies for children with UCP.

## Conclusion

Preschool-aged children with UCP prioritized self-care and leisure goals, reflecting developmental needs and caregiver perspectives. Goals related to dressing and quiet leisure were associated with greater functional improvement after CIMT. Understanding these patterns can help occupational therapists design more individualized, developmentally appropriate interventions for young children and their families.

## Supporting information

S1 Table Goal importance scores (mean ± SD) by MACS group (I–II vs. III). (DOCX)
