# Supplementary material for: Personalized goal setting and predictors of functional gains following constraint-induced movement therapy in preschool-aged children with unilateral cerebral palsy
Source: PLoS One. 2025 Aug 6;20(8):e0329002. doi: 10.1371/journal.pone.0329002 (PMC12327683; doi:10.1371/journal.pone.0329002)
Supplement: S1 Table — (DOCX) [file pone.0329002.s001.docx]

**Supplementary Material**

**Personalized goal setting and predictors of functional gains following constraint-induced movement therapy in preschool-aged children with unilateral cerebral palsy**

| **S1 Table.** Goal Importance Scores (Mean ± SD) by MACS Group (I–II vs. III) | | | | |
| --- | --- | --- | --- | --- |
|  | **MACS I–II** | | **MACS III** | |
|  | **N** | **Mean (SD)** | **N** | **Mean (SD)** |
| **Dressing** | 14 | 9.00 (1.16) | 5 | 8.26 (1.25) |
| **Eating** | 12 | 8.58 (1.53) | 5 | 8.20 (1.30) |
| **Bathing** | 9 | 6.67 (0.86) | 3 | 6.33 (0.57) |
| **Hygiene** | 13 | 8.15 (1.07) | 5 | 8.60 (0.89) |
| **Quiet Leisure** | 13 | 8.20 (1.64) | 4 | 6.80 (1.92) |
| MACS, Manual ability classification system | | | | |
